# Ovarian cancer, the coagulation pathway, and inflammation

**DOI:** 10.1186/1479-5876-3-25

**Published:** 2005-06-21

**Authors:** Xipeng Wang, Ena Wang, John J Kavanagh, Ralph S Freedman

**Affiliations:** 1 Department of Gynecologic Medical Oncology, The University of Texas M. D. Anderson Cancer Center, Houston, TX, USA; 2 Department of Transfusion Medicine, National Institutes of Health, Bethesda, MD, USA; 3 Department of Gynecologic Oncology, The University of Texas M. D. Anderson Cancer Center, Houston, TX, USA

## Abstract

Epithelial ovarian cancer (EOC) represents the most frequent cause of death in the United States from a cancer involving the female genital tract. Contributing to the overall poor outcome in EOC patients, are the metastases to the peritoneum and stroma that are common in this cancer. In one study, cDNA microarray analysis was performed on fresh tissue to profile gene expression in patients with EOC. This study showed a number of genes with significantly altered expression in the pelvic peritoneum and stroma, and in the vicinity of EOC implants. These genes included those encoding coagulation factors and regulatory proteins in the coagulation cascade and genes encoding proteins associated with inflammatory responses. In addition to promoting the formation of blood clots, coagulation factors exhibit many other biologic functions as well as tumorigenic functions, the later including tumor cell proliferation, angiogenesis, invasion, and metastasis. Coagulation pathway proteins involved in tumorigenesis consist of factor II (thrombin), thrombin receptor (protease-activated receptors), factor III (tissue factor), factor VII, factor X and factor I (fibrinogen), and fibrin and factor XIII. In a recent study we conducted, we found that factor XII, factor XI, and several coagulation regulatory proteins, including heparin cofactor-II and epithelial protein C receptor (EPCR), were also upregulated in the peritoneum of EOC.

In this review, we summarize evidence in support of a role for these factors in promoting tumor cell progression and the formation of ascites. We also discuss the different roles of coagulation factor pathways in the tumor and peritumoral microenvironments as they relate to angiogenesis, proliferation, invasion, and metastasis. . Since inflammatory responses are another characteristic of the peritoneum in EOC, we also discuss the linkage between the coagulation cascade and the cytokines/chemokines involved in inflammation. Interleukin-8, which is considered an important chemokine associated with tumor progression, appears to be a linkage point for coagulation and inflammation in malignancy. Lastly, we review findings regarding the inflammatory process yielded by certain clinical trials of agents that target members of the coagulation cascade in the treatment of cancer. Current data suggest that disrupting certain elements of the coagulation and inflammation processes in the tumor microenvironment could be a new biologic approach to cancer therapeutics.

## Introduction

An estimated 22,220 new cases of and 16,210 deaths from epithelial ovarian cancer (EOC) will occur in 2005 [1]. EOC is insidious: 75% of patients present at an advanced stage, in which metastasis to the peritoneal cavity lining is characteristic. Peritoneal and serosal involvement is associated overall with a poor patient outcome. The mechanism of peritoneal seeding, spreading, and progression is poorly understood. Direct contact of free-floating ovarian tumor cells in the peritoneal cavity may place the peritoneum at risk for metastatic spread. Other evidence of metastatic spread points to a surface epithelium origin of EOC [2] or genetic instability of the stroma [3].

Evidence is mounting that an inflammatory process contributes to tumor growth and metastasis to the peritoneum in EOC [4,5]. This process is facilitated by cross talk between tumor cells and the surrounding cellular stroma [6]. Surgical findings from EOC frequently include enhanced tumor vascularity with or without malignant ascites. Microscopic examination may reveal an inflammatory infiltrate comprising different leukocyte populations [6]. The stroma associated with tumors may resemble granulation tissue formed during wound healing [7]. The interaction of cancer cells with their surroundings might influence phenotypic alterations in the tumor. For example, the presence of human breast tumor stromal explants in mice may determine the formation of breast cancer-like lesions [8].

In a recent study, we compared the transcriptional profile of normal-appearing peritoneum and its adherent and subjacent stroma in EOC patients with that of patients with pathologically benign ovarian disease [3]. In our study, we identified 402 genes differentially expressed between malignant and benign peritoneum and 663 genes differentially expressed between malignant and benign stroma. No significant differences in gene expression between malignant peritoneum and malignant stroma were observed, which suggests that the transcriptional changes induced by the malignant condition overlapped at least partly. Our preliminary analysis indicated that the gene profile of peritoneal structures might be affected by the presence of EOC cells [3]. We found it interesting that the genes included those linked to inflammatory processes and a number were linked to the coagulation pathway. An examination of these two processes at the gene transcript level suggests a number of possible links between coagulation and inflammation. In this paper, we discuss the different roles of coagulation factor pathways as they relate to angiogenesis, proliferation, invasion, and metastasis in the tumor and peritumoral microenvironment.

### Coagulation and cancer development

The history of a known association between coagulation and cancer dates back to 1865, when Armand Trousseau observed that patients who presented with idiopathic venous thromboembolism frequently had an underlying occult cancer and vice versa [9]. The association has only recently become more apparent. Previously, researchers focused on how tumors activate blood coagulation and how to overcome it. However, the underlying mechanism by which coagulation factors promote tumor cell growth, invasion, metastasis, and angiogenesis has become a hot topic in the field of cancer research. Some coagulation factors that displaya role in tumor progression have been reviewed [10]. The most frequent reports on coagulant proteins and cancer interactions include factor III (tissue factor; TF) [11], TF-factor VIIa [12,13], factor Xa [14], factor IIa (thrombin)-factorII receptors (also called proease-activated receptors (PARs) [15], and factor XIIIa-factor Ia (fibrin) (Table 1) [16]. Two cascades, one intrinsic and the other extrinsic, lead to the formation of a fibrin clot. Although they are initiated by distinct events, the two cascades converge on a common pathway that includes thrombin, PARs, and fibrin and lead to clot formation. The intrinsic cascade, which is initiated when contact is made between blood and exposed endothelial cell (EC) surface, requires the clotting factors VIII, IX, XI, and XII. Also required are the proteins prekallikrein and high-molecular-weight kininogen and Ca^2+ ^and phospholipids secreted from platelets. The extrinsic coagulation cascade is initiated at the site of injury in response to the release of TF. Activated factor X is the site at which the intrinsic and extrinsic coagulation cascades converge. TF and factor VIIa contribute to the extrinsic cascade and possibly to the development of cancer. Other factors from the intrinsic pathway, such as factors XI and XII, have not yet been directly implicated in cancer progression. Our recent analysis of the gene expression profile in EOC has revealed increased levels of factor XI and factor XII transcripts in the peritoneum of EOC patients [3]. These findings suggest that the intrinsic cascade might promote ovarian cancer cell metastasis in the peritoneal cavity. We also showed that the serine proteinase inhibitor D1 (SERPIND1), which inhibits plasmin, tissue kallikrein, and factor XIa, were downregulated [3].

**Table 1:** Coagulation factors and the associated regulatory proteins in EOC

**Coagulation factors**	**Effects**
Tissue factor (TF) ↑	Promoting angiogenesis by activation of MAPK[73] and protein C kinase C-dependent signaling[76]; TF-PAR2 selectively synergizes with PDGF-BB to enhance to metastasis in lymphnodes[78]. Promoting invasion and metastasis by the activation of P21^Ras ^and P42/P44 MAPK pathway to inhibit apoptosis[14]; overexpressing growth factors and chemokines (i.e. IL-8)[13].
TF-VII-PAR2 ↑	Promoting angiogenesis, invasion, and metastasis by clotting-independent mechanism[77] in presence of inflammatory cytokines
Factor X ↑	Forming complex with TF-VIIa to promote tumor angiogenesis and metastasis[14]
Thrombin/PAR1 ↑	Promoting angiogenesis by inhibiting EC migration to collagen type IV or to laminin[99]; upregulating VEGF expression[100]. Promoting invasion and metastasis depended on at least 6 mechanisms (text)
Fibrinogen/fibrin ↑	Stimulating angiogenesis; the fibrin gel matrix facilitating tumor metastasis; increasing plasma exudates to form ascites[129, 130, 132].
Factor XII/XI ↑	Positive feedback on human kallikreins system
Factor XIII ↑	Form stable fibrin
	
**Regulatory proteins**	
Heparin cofactor II ↑	Produce chemoattractant peptide for MAs migration.
Endothelial protein C receptor ↑	Intensifying APC-PAR1 signal transduction [205] and contributing to antiapoptosis in tumor.
Tissue factor pathway inhibitor ↓	Loss of control of tumor growth and metastasis by activating Factor Xa and increasing Factor Xa-PAR2 signaling[81].
Tissue factor pathway inhibitor-2 ↓	Loss of Inhibiting TF-VIIa complex and various protease but not Factor Xa; Loss of antiangiogenesis and antimetastasis.

Blood coagulation cascades can be activated by different mechanisms and to different levels in cancer patients. The alterations range from subtle abnormalities in laboratory tests to clinically overt thrombosis and disseminated intravascular coagulation [17]. Up to 50% of all cancer patients and 90% of those with metastases exhibit hemostatic abnormalities [17]. These abnormalities may be reflected in the dominance of the tumor cell-associated procoagulant pathway, which leads to thrombin generation and hypercoagulation. Similar observations were made using *in vitro *ovarian cancer cells for the coagulation process. Ovarian cancer cells may also express TFs and the other coagulation components that generate local thrombin, as indicated by the conversion of fibrinogen to fibrin [18]. Fibrin has been found on ECs and the surface of ovarian tumor cells and nodules [18]. Coagulation activation in malignancy may be triggered by direct or indirect mechanisms. Direct activation of blood coagulation by the induction of thrombin may occur through the activity of tumor cell procoagulation, whereas indirect activation may occur through the production of tumor-associated cytokines that trigger TF production by host macrophages (MAs) or ECs. The coagulation pathway components may contribute to tumor cell proliferation, invasion, and metastasis [10], although these alterations could also be a consequence of advanced disease [19].

#### Factor XII and the kallikrein family (positive feedback loop)

Factor XII is a procoagulant protein that participates directly or indirectly in activating the intrinsic clotting pathway. The structure of factor XII, which has been inferred from its DNA and amino acid sequences, include two epidermal growth factor (EGF) homologous domains in the amino-terminal region [20]. This type of structure suggests that factor XII might mimic EGF biological characteristics and act as a growth factor. One study showed that factor XII had mitogenic effects on the HepG2 human hepatocellular carcinoma cell line and, similar to EGF, stimulated tumor cell proliferation [20].

Assembly of contact phase components results in the conversion of prekallikrein to kallikrein, which in turn activates factor XII to factor XIIa. Factor XIIa then hydrolyzes more prekallikrein to kallikrein, thereby establishing a reciprocal activation loop. Factor XIIa also activates factor XI to factor XIa and leads to the release of bradykinin, a potent vasodilator, from high-molecular-weight kininogen. The human kallikreins (hK) are a subfamily of the serine protease enzyme family [21], which consists of proteolytic enzymes important to various physiologic processes, such as digestion, coagulation, fibrinolysis, apoptosis, cell migration, tissue remodeling, and inflammation [21]. The hK gene family, which includes 15 members (hK1-hK15) clustered in a 300-kb region on chromosome 19q13.4, could be altered in cancer. A number of studies have reported increased amounts of kallikrein transcripts or proteins in cancer cells, particularly adenocarcinomas derived from steroid hormone-regulated tissues [21]. At least 11 kallikrein genes or proteins have been found to be overexpressed in EOC; they are hK4 [22], hK5 [23,24], hK6 [25,26], hK7 [27], hK8 [28,29], hK9 [30], hK10 [31-33], hK11 [34-37], hK13 [38,39], hK14 [40,41], and hK15 [42] mRNA or protein in ovarian cancer tissue, cell lines, serum, and tumor ascites fluid (Table 2). A recent gene microarray analysis on ovarian cancer tissue found overexpression of hK2 and hK3 [43]. Overexpression of hK8 [28,29], hK9 [30], hK11 [34,35], hK13 [38,39], and hK14 [41] have been reported as independent variables associated with longer progression-free and overall survival. Another study found hK11 to be an unfavorable factor for EOC [36]. The other hKs have all been linked to progression in EOC (Table 2). Although many hKs are overexpressed in EOC tissues and in ascites, it is unknown whether this phenomenon reflects increased proteolytic activity because of the heterogeneity of hK forms and the presence of active enzymes found in the extracelluar matrix (ECM). This apparent paradox in which the clinical outcome differs with different hKs might be due to different biologic roles of hKs in the tumor progression process, either stimulating or inhibiting the development of the tumor and its microenvironment. hK-mediated pericellular proteolysis in the ECM might help regulate tumor cell growth, angiogenesis, invasion, and metastasis, which could contribute to tumor progression [21]. Some hKs are under steroid hormone regulation and might represent downstream targets through which hormones affect the initiation or progression of EOC.

**Table 2:** Expression and clinical features of hK members in ovarian cancer

**hK family member**	**Location**	**Expression level and site**	**Clinical feature**	**Prognosis**
hK4	Tumor cells	Increased in EOC tissue	Predictive marker for paclitaxel resistance[22]	Unfavorable
hK5	Serum, ascites, and tumor extracts [23, 24]	Increased on tumor cells and in serum and ascites	Potential biomarker for diagnosis	Unfavorable
hK6	Tumor cell[26] and serum[25]	High in early-stage and low-grade tumor tissue and in EOC serum	Overexpression is an early phenomenon in the development of ECO; serum level could be used as a biomarker	Unfavorable
hK7	Tumor tissue[27]	High in late-stage EOC	A potential biomarker for diagnosis	Unfavorable
hK8	Tumor extract, serum, and ascites[29]	High in serum and ascites	High level is associated with good prognosis[28, 29]	Favorable
hK9	Tumor cells[30]	High in tumor tissue early-stage and optimal debulking patients	Associated with longer progression-free and overall survival times	Favorable
hK10	Serum[33] and tumor cells[31,32]	High in serum and on tumor cells	High serum level is associated with increased risk for relapse and death; a potential biomarker for diagnosis	Unfavorable
hK11	Serum, ascites[37], and tumor extract [34-36]	Increased in tumor samples	High level is an independent factor for favorable prognosis and is associated with long progression-free and overall survival times and with slower disease progression[34, 35]; however, high level is also associated with poor survival rate[36].	Depends
hK13	Tumor tissue[38]	verexpressed in tumor tissue	Associated with longer progression-free and overall survival times	Favorable
hK14	Tumor cells[41] and serum[40]	Increased in tumor tissue and serum	Associated with longer progression-free and overall survival times; an independent prognostic factor	Favorable
hK15	Tumor extract[42]	Increased in tumor tissue	Associated with short progression-free and overall survival times; an independent prognostic factor	Unfavorable

Other data have shown that hK5-hK7 can contribute to ECM remodeling through fibrinogen, collagen types I and IV, laminin, and fibronectin and, in this fashion, facilitate tumor angiogenesis, invasion, and metastasis [21]. Kallikreins might also promote angiogenesis by disrupting ECM barriers. hK2 [44], hK3 [45], hK6 [46], hK7 [27], and hK14 [41] directly catalyze the hydrolysis of certain ECM proteins, enabling both EC and tumor cell migration and invasion. The ECM may also be remodeled through activation of the uPA-PAR system [21]. For example, hK2 and hK4 may remodel certain ECM components through plasmin and release or activate certain pro-angiogenic growth factors such as vascular endothelial growth factors (VEGFs) and pro-matrix metalloproteins (MMPs) [47]. It could be assumed that factor XII and kallikrein family members might also participate together in the formation of ascites and peritoneal implants in EOC. Furthermore, hK1 is expressed in ECs and hK1-generated kinins, which are multifunctional, biologically active peptides released from low-molecular-weight kininogen that can stimulate angiogenesis [48]. The remodeling effect of hKs on ECs has been demonstrated in Matrigel *in vitro *invasion assays. For example, invasion of MDA-MB-231 human breast cancer cells into Matrigel was suppressed by a synthetic hK1 inhibitor [49], and invasion of LNCaP human prostate cancer cells through Matrigel was attenuated by hK3-neutralizing antibodies [45]. Conversely, hK3 has been shown to have antimetastatic properties in mice [45]. The antimetastatic effect might be due to an antiangiogenic effect, which is independent of serine protease action. hKs might also be able to promote cancer cell invasion through PAR signaling in a manner similar to thrombin [50].

According to a study conducted by Rehault, hK2 and hK3 could represent important regulators of insulin-like growth factors (IGFs) in the proliferation of prostate cancer cells [51]; however, similar findings have not been published on EOC. IGFs are mitogenic peptides that help regulate normal and malignant cellular proliferation, differentiation, apoptosis, and transformation. IGFs have to be released from IGF-binding proteins (IGFBPs) before their activation. hK1, hK2, and hK3 are IGFBP proteases that collectively degrade IGFBPs 2–5 and could abrogate their affinity for IGF1. In our microarray analysis of the peritoneum of EOC patients, we detected overexpression of IGF1 and decreased expression of SPINT2. These findings are important because SPINT2 inhibits the production of tissue kallikreins [3]; therefore, it could be deduced that a high expression of hKs might result. In the meantime, IGF1 can stimulate the growth of normal, stromal, and malignant prostate cells *in vitro *[51,52]. The IGF-IGFBP systems can operate in many organs, suggesting roles for them in cancer growth [53].

The blood coagulation and kallikrein-kinin systems consist of plasmatic proteolytic cascades. These cascades participate in the initial host response against foreign components and in wound healing. The increased expression of factor XII in the peritoneum tissue of EOC patients [3] suggests that the intrinsic coagulation cascade is being activated and may be involved in ovarian carcinoma cell progression. At least two mechanisms may be responsible for activating the cascade: the molecular structure of factor XII mimics the EGF motif and, thus, may have a growth factor-like role on cancer cells, and because of the positive feedback loop, the kallikrein system is capable of promoting ovarian cancer cell growth, angiogenesis, metastasis, and invasion.

#### Factor XI

Factor XI is a component of the intrinsic coagulation pathway and is activated after factor XII activation. Factor XI is the zymogene (i.e., the gene associated with a proteolytic enzyme) precursor of a plasma serine protease produced primarily in the liver that contributes to blood coagulation by activating factor IX by limited proteolysis [54]. Factor XI mRNA has been detected in human platelets [55], but a link between factor XI and the stimulation or inhibition of cancer cell proliferation and metastasis has not been established. In our microarray analysis of the peritoneum of EOC patients, we found a higher expression of factor XI mRNA in peritoneum and stroma tissue in the general vicinity of ovarian cancer implants [3]. Because factor XII expression also increased, factor XI might be induced by the activation of factor XII. In addition, factor XI might have an indirect role in the pathogenesis of cancer by facilitating downstream factors in the coagulation cascade, such as thrombin and its receptors. These are discussed later.

#### Tissue factor

There is abundant evidence that TF plays an important role in the pathogenesis of cancer. Human TF molecule is a single-chain, 263-amino-acid, 47-kDa transmembrane glycoprotein whose primary sequence indicates structural similarity to members of the cytokine receptor family [56]. TF is the principal surface receptor and cofactor for the activated coagulation protease factor VIIa and is a receptor for its zymogen precursor. The extrinsic coagulation cascade is triggered by the binding of factor VIIa to TF, which creates a complex for the activation of the protease factor X and its conversion to factor Xa. Generation of factor Xa and the TF-factor VIIa complex triggers the proteolytic conversion of factor II (prothrombin) to thrombin.

TF is expressed constitutively on the adventitia of uninjured blood vessels and other extravascular tissues [57]. Upregulation of TF gene expression occurs in malignant cells and in normal host cells that respond to inflammatory or remodeling signals, which might arise from ECs, MAs, and fibroblasts [58]. Therefore, cytokines and growth factors, such as platelet-derived growth factor (PDGF), fibroblast growth factor (FGF), transforming growth factor-β (TGF-β), and EGF [58], produced by inflammatory and malignant cells might induce the expression of TF in fibroblasts and ECs. Experimental evidence has also shown that expression of TF by ECs can be under the control of VEGF, which can be mediated by the VEGF receptor fms-like tyrosine kinase (flt-1) and flt-1/kinase insert domain-containing receptor [59]. Two downstream components of the coagulation cascade, thrombin [60] and fibrin [61], can also regulate TF expression by ECs. Both downregulation and inhibition of TF expression can modulate tumor cell procoagulant activity. Recent evidence suggests that TF expression might alter the cancer cell phenotype and possibly contribute to angiogenesis, proliferation, and metastasis [62-69]. Aberrant TF expression has been detected in various human tumors, including EOC [62-64], breast cancer [65], non-small cell lung cancer [66], colon cancer [67], and pancreatic cancer [68] but is not usually found in normal tissues from these sites. Elevated expression of TF in tumors has been associated with certain unfavorable prognostic indicators, such as angiogenesis, metastasis, advanced disease stage, and multidrug resistance [69].

### TF and angiogenesis

The formation of new cellular masses at an organ site creates an obvious extra demand for oxygen, growth factors, and metabolites and increases the need for tumor-associated blood vessels. The stimulators and inhibitors of angiogenesis that can regulate new vessel formation include VEGF, acidic and basic FGF, hepatocyte growth factor, and TGF. Angiostatin, endostatin, and antiangiogenic antithrombin might function as inhibitors of angiogenesis [70].

Angiogenesis can be mediated by either coagulation-dependent or-independent activation pathways [71]. Coagulation-dependent pathways always involve activation of the TF receptor to its ligand, followed by production of thrombin and ensuing clot formation. Coagulation-independent pathways appear to involve phosphorylation of the cytoplasmic domain of the TF receptor and subsequent downstream signaling events that occur independently of thrombin production or clot formation. Both pathways can contribute to angiogenesis and tumor progression. Increased expression of TF in tumors may contribute to angiogenesis in part by increasing VEGF protein expression and downregulating the expression of the antiangiogenic protein thrombospondin [11]. For example, TF-positive colorectal tumors have higher levels of microvessel density and VEGF expression than TF-negative colorectal tumors do [67]. Colocalization of TF and VEGF mRNA and protein has been demonstrated in breast cancer, malignant glioma, and adenocarcinoma of the lung [72]. In the coagulation-dependent pathway, the interaction of TF and factor VIIa induces Ca^2+ ^oscillations and changes in gene expression, and the formation of the TF-factor VIIa complex leads to the activation of the mitogen-activated protein kinase (MAPK) pathway [73], which is a major inducer of VEGF expression. The catalytic activity of the TF-factor VIIa complex contributes to the activation of factor X to factor Xa and subsequently to thrombin. Factor Xa and thrombin) can also exert signaling activities through PAR-2 and PAR-1, respectively. Factor Xa activated by tumor cells may trigger PAR-2 expression as well as formation of TF-factor VIIa complexes on ECs and may induce intracellular signals; however, factor Xa exerts the most critical effect in the TF-factor VIIa-factor Xa complex [74].

In the clotting-independent pathway, the cytoplasmic domain of TF activates protein kinase C-dependent signaling, in contrast to the ligand-binding extraplasmatic tail of TF, which upregulates the synthesis of VEGF in response to different stimuli [75,76]. The cytoplasmic tail of TF appears to regulate non-clotting-dependent mechanisms such as cytoskeletal reorganization, vascular remodeling, angiogenesis, and cellular metastasis. Results from a recent study provided an alternative explanation whereby TF can promote angiogenesis by a clotting-independent mechanism [77]: the genetic deletion of the TF cytoplasm domain involves the loss of negative regulatory control with PAR-2 upregulation. Expression of TF and PAR-2 and the function of TF-PAR-2 signaling pathway require the presence of both angiogenic growth factors and inflammatory cytokines. Inflammatory cytokines produced by monocytes (MO) are important for angiogenesis and collateral growth vessels. Colocalization of upregulated PAR-2 with phosphorylated TF occurs only in neovessels. The phosphorylation of TF appears to be the mechanism that switches off the negative regulatory control that promotes pathologic PAR-2-dependent angiogenesis [77].

However, TF-PAR-2 signaling selectively synergizes with the PDGF isoform BB but not with VEGF, basic FGF, and the PDGF isoform AA. PDGF-BB is available through release from activated platelets in the context of local coagulation or by synthesis from sprouting ECs. PDGF-BB also has a role in lymphoangiogenesis. PDGF-BB stimulated MAPK activity and cell motility of isolated lymphatic ECs *in vitro *and potently induced the growth of lymphatic vessels *in vivo *[78]. Expression of PDGF-BB in murine fibrosarcoma cells induced tumor lymphoangiogenesis, leading to enhanced metastasis in lymph nodes [78].

The cascade of TF-factor VIIa to factor Xa and subsequently to thrombin can be inhibited by TF pathway inhibitor (TFPI), which occurs when the TF-factor VIIa complex is combined with factor Xa and cell-bound TFPI. Once this stable quaternary complex is formed, it is translocated to and internalized in caveolae (i.e., small vesicles of the plasma membrane) [79]. Produced by ECs and tumor cells, most TFPI is expressed on the cell surface, although it can be detected peripherally in plasma [80]. In tumor cells, the diminished expression of TFPI could result in activated factor Xa and increase factor Xa-PAR-2 signaling [81]. Results from *in vitro *and *in vivo *studies have suggested that therapeutic strategies that target an increase in the expression of TFPI could inhibit tumor angiogenesis [82], growth [83], and metastasis [53,84].

Another inhibitor of the TF-dependent pathway of blood coagulation that was recently identified is TFPI-2, which, in contrast to TFPI, inhibits the TF-factor VIIa complex but not factor Xa. TFPI-2 also inhibits various proteases [85]. Its expression was lower in laryngeal, breast, gastric, colon, pancreatic, renal, and endometrial tumor tissues and glial neoplasms than in normal counterpart tissue [85]. Some studies have provided evidence of the antiangiogenic and antimetastatic activity of TFPI-2 [86]. Epigenetic inactivation of TFPI-2 also contributes to the progression of pancreatic ductal adenocarcinoma. TFPI-2 mRNA was undetectable in many pancreatic cancer cell lines and in primary pancreatic ductal neoplasms. Hypermethylation of its promoter CpG island decreased TFPI-2 expression. However, expression of the TFPI-2 gene in pancreatic cancer cells has resulted in markedly suppressed proliferation, migration, and invasive potential *in vitro *[86]. TFPI-2 exerts antitumor effects through antiangiogenesis and antimetastatic mechanisms [87,88].

### TF and metastasis

The role of TF-factor VIIa in metastasis has been extensively investigated. Some recent studies have revealed that TF can promote the invasion and metastasis of the A2780 human ovarian cancer cell line through the TF-factor VIIa pathway. In turn, TF-factor VIIa can upregulate the transcription of uPA receptor expression and enhance tumor invasion and metastasis [63,64]. Overexpression of TF of up to a 1,000-fold greater level than normal is characteristic of metastatic tumor cells relative to nonmetastatic cells [89]. Inhibition of TF through antibodies or active site-blocked factor XIIa interferes with experimental metastasis [90], but how TF on tumor cells contributes to tumor metastasis is unclear. TF-induced metastasis requires the participation of the cytoplasmic tail of TF and the assembly of an active TF-factor VIIa complex, which is thought to target G protein-coupled PAR-2. Factor VIIa induces the activation of p21^Ras ^and p42/p44 MAPK, which further induces the activation of the protein kinase B pathway in TF-expressing cells and increases protein synthesis [91]. Both the p42/p44 MAPK and protein kinase B pathways are able to inhibit apoptosis. Inhibition of apoptosis may be related to adhesion-independent survival and thus may be linked to metastasis. In one study, factor VIIa inhibited cell death and caspase 3 activation in Baby Hamster Kidney cells induced by serum-deprivation and loss of adhesion (lack of integrin signaling) when transfected with TF genes [14]. The effects of factor VIIa on caspase 3 activity are sensitive to inhibitors of the phosphatidylinositol 3'-kinase and P42/P44 MAPK pathways; thus, factor VIIa appears to be a survival factor for TF-expressing cells and seems to mediate the survival of tumor cells during metastasis [14,92].

**Figure 1:**
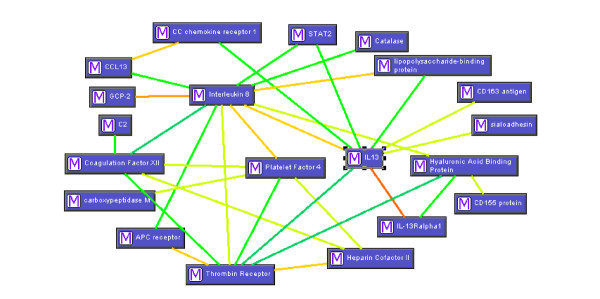
Genes associated with MO/MA activation. This figure was made by cellspace software according to an analysis of the functional genes involved in inflammatory response in peritoneal cavity with EOC[3]. The connected lines with different colors show different levels of association among genes. The warmer colors (yellow to red) represent stronger correlations.

Another mechanism that links TF effects to metastasis is mediated by the overexpression of growth factors and proteins related to cellular reorganization. Interleukin (IL)-8, a member of the CXC chemokine (CXCL8) and a chemoattractant for neutrophils and lymphocytes, can act multifunctionally to induce tumor growth and metastasis, and IL-8 overexpression can occur through TF-FVIIa stimulation. In contrast, FXa and thrombin cannot upregulate IL-8 expression. IL-8 leads to increasing cell migration and invasion, and these effects are attenuated when binding of factor VIIa to TF is prevented [13]. In ovarian cancer studies, IL-8 has been suggested to be involved in the formation of ascites [93,94]. In our peritoneal gene transcript profile, we showed that IL-8 connects a number of pathways that might be linked to the pathogenesis of cancer by cellspace analysis (Figure 1) [3]. IL-8 may induce other immune cell-related modulators, such as the IL-13 receptor, vascular cell adhesion molecule 1, chemokine receptor 1, and other molecules associated with inflammatory function [95]. It is known that the inflammatory response is involved in the progression of tumors and that IL-8 might amplify inflammation in the tumor microenvironment to promote tumor cell proliferation, angiogenesis, and metastasis.

#### Thrombin and thrombin receptor

Generation of thrombin is the central step in blood coagulation. Expression of thrombin has been detected in various tumor types, including ovarian cancer [18]. Thrombin's cellular effect is triggered by its binding to PARs, which are G protein-coupled receptors, of which four subtypes have been identified in human tissues. PAR-1, PAR-3, and PAR-4 are activated mainly by thrombin, whereas PAR-2 is activated by trypsin, tryptase, factor VIIa, and factor Xa. PAR-1, the major prototype of the family, is a receptor with seven transmembrane domains and is activated by cleavage of a specific site at the N-terminus of its extracellular domain. The presence of PAR family members has been demonstrated on many tumor cells, and it appears to be positively correlated with metastasis [96]. Studies of proteins at the cellular level (*in situ *hybridization and immunostaining) and studies of gene transcripts (reverse-transcriptase polymerase chain reaction) have demonstrated the differential expression pattern of PAR-1 gene in low-grade and high-grade EOC, but PAR-1 expression has not been shown on normal ovarian tissue even though the gene was overexpressed in the peritoneum of EOC patients [3,97]; thus PAR-1 signaling could be involved in the initiation and pathogenesis of EOC.

### Thrombin-PAR system and angiogenesis

Thrombin is a potent activator of angiogenesis and could be the underlying mechanism for the promotion of tumor progression after thrombosis. However, evidence suggests that angiogenesis induced via thrombin is mediated via PAR and is independent of fibrin formation, which can be modulated without interfering with blood coagulation [98]. Thrombin also stimulates the chemotaxis of inflammatory cells and thus possibly promotes tumor angiogenesis indirectly [98]. Furthermore, thrombin could induce several cellular effects on ECs, thereby contributing to the angiogenic cascade.

There are two possible mechanisms by which thrombin may promote angiogenesis: (1) Thrombin at physiologic concentrations can inhibit ECs' ability to adhere to collagen type IV or to laminin [99], even after short exposure of ECs to thrombin. The result is reversible and is mediated specifically through the thrombin receptor. The result is detachment and migration of ECs from basement membrane components, the initial step in the activation of normally quiescent ECs. (2) Thrombin upregulates VEGF expression via PAR activation on ECs or tumor cells [100] and through release of platelets [101]. Thrombin antagonizes any blood vessel-stabilizing effects of angiopoietin 1 by production of angiopoietin 2 [102,103]. The angiogenic effects of thrombin include causing the release of basic FGF from ECs and the ECM [104]. Thrombin can also markedly stimulate expression of the VEGF receptor (kinase insert domain-containing receptor and flt-1). The activation of protein C kinase and MAPK signaling pathways was confirmed in the process of VEGF and VEGF receptor production [102]. Thrombin may thus contribute to new vessel formation by providing the pathologic microenvironment for tumor cell implants and proliferation; VEGF production through thrombin activation could facilitate this process. VEGF also increases the permeability of existing and new capillary vessels and results in plasma protein leakage and the development of a proangiogenic matrix [105]. A high expression level of VEGF allows the formation of malignant ascites. Thus, the thrombin-PAR system could play a role in the progression of ascites in EOC patients by inducing VEGF production.

### Thrombin-PAR system in invasion and metastasis

In our peritoneal transcript profile study, we found that PAR-1 was upregulated in peritoneal tissue, which suggested that this receptor might be involved in EOC pathogenesis, invasion, and metastasis [3]. Overexpression of PAR-1 has been reported in malignant invasive melanoma [106] and breast cancer *in vivo *[96] and in breast cancer cell lines [107]. The increase in growth and metastatic potential of tumor cells may be partly attributable to the proangiogenic effects of thrombin. By mobilizing adhesion molecules, such as α_II_β_3 _integrin [108], P-selectin [109], and CD40 ligand [110], to the cell surface, thrombin enhances adhesion between tumor cells, platelets, ECs, and ECM and contributes to tumor progression. Thrombin also triggers the release of growth factors, chemokines, and ECM proteins that could promote the proliferation and migration of tumor cells. Facilitation of the metastatic activity of the thrombin-PAR complex has been demonstrated *in vivo *with experimental lung cancer [111], colon cancer [112], and breast cancer [15,96]. The principal thrombin receptor, PAR-1, has been implicated in promoting these effects. Most of the cellular effects elicited by thrombin are mediated through the activation and subsequent signal transduction cascades of members of the PAR family. Likewise, thrombin and PAR inhibitors, such as argatroban [113], activating protein 1 [112], and small interfering RNAs for PAR-1 [114], have been shown to prevent tumor migration and metastasis. Thrombin can also facilitate trans-basement membrane migration by ECM remodeling after activation of collagen type IV-degrading enzyme, MMP-2, and α_v_β_3 _integrin. However, an immunostaining study on EOC found that focal adhesion kinase (FAK), a major tyrosine phosphorylated protein of adherent cells, was abundantly expressed in invasive EOC but not in normal ovarian tissues, which is consistent with a high level of PAR1 expression [97]. Possibly, PAR1 in malignant ovarian carcinoma transmits signals leading to the phosphorylation of FAK and thereby to alterations in integrin expression. Increased expression of α_v_β_3 _integrin was also detected in our peritoneal profile study [3]. Anticoagulants such as heparin and warfarin have been shown to significantly decrease experimentally induced pulmonary metastasis *in vivo *[115], and a similar decrease in tumor cell metastasis was observed *in vivo *with hirudin, a highly specific inhibitor for thrombin [15]. In another study, hirudin inhibited tumor implantation and growth of four different tumor cell lines (MDA-MB-231 human breast cancer, 4T1 human breast cancer, A549 human non-small cell lung cancer, and B16F10 murine melanoma) in nude and syngeneic mice by inhibiting spontaneous seeding of tumors through the blood to susceptible organs, including the lungs, liver, spleen, and lymph nodes [15]. Overall, the interaction of endogenously generated thrombin and host thrombin receptors enhances tumor angiogenesis by enhancing the expression of some genes that may be associated with an invasive profile, and this interaction involves activation of PAR and other tumor genes.

At least six signal transduction mechanisms based on thrombin-PAR systems have been proposed to have a role in invasion and metastasis: (1) PAR may induce phosphorylation of FAK and integrin expression [97,116], thereby contributing to the modulation of cytoskeletal reorganization. Changes in cell shape result from changes in actin stress fibers and focal adhesion and are mediated through the Rho family of GTPases [117]. Rho-Ras proteins downstream of PAR-1 are intermediates of the extracellular signal-regulated kinases 1 and 2 (ERK1/2)-MAPK pathway and are implicated in cancer progression [118]. (2) Loss of activator protein 2 (AP-2) results in the increased expression of PAR-1, which subsequently contributes to the metastatic phenotype of cancer by upregulating the expression of angiogenic molecules, proteases, and adhesion molecules, which are involved in tumor invasion and metastasis [119]. This upregulation is based on MAPK signaling. AP-2 is a 52-kDa protein transcription factor mapped to the short term of chromosome 6, which is a critical regulator of gene expression during mammalian development, differentiation, and carcinogenesis. Loss of AP-2 may contribute to the development and progression of many different types of cancers, including melanoma and prostate, breast, and colorectal carcinoma [119]. AP-2 is a negative regulator for PAR-1 signaling, and functional AP-2-binding elements have been identified in some genes, including c-erbB2, VEGF, TGF-β, IGFBP-2, E-cadherin, and c-myc [119]. (3) Overexpression of flotillin-2, a highly conserved caveolae/lipid raft-associated protein, is associated with upregulation of PAR-1. Binding of flotillin-2 to PAR-1 might stabilize it by blocking inactivation or degradation of an activated PAR-1. Cytoplasmic flotillin-2 might also modulate PAR-1 trafficking of the cell or stabilize PAR-1 mRNA, which could promote PAR-1-induced constitutive signaling through the MAPK or other signal transduction pathways [120]. (4) PAR-1 cooperates with α_v_β_5 _integrin to promote cytoskeletal reorganization. Activation of PAR-1 increased the phosphorylation of FAK and paxillin and induced the formation of focal contact complexes. The activation of PAR-1 may foster tumor cell invasion via a mechanism involving cooperation with the α_v_β_5 _integrin [116]. (5) The increased and persistent expression of PAR-1 is induced by deregulated defective receptor trafficking, which is correlated with the failure to efficiently downregulate activated PAR-1 by internalization and lysosomal sorting [121]. (6) Coexpression of PAR-1 and PAR-2 has been observed on some tumor cells and the cells of the tumor microenvironment, such as fibroblasts, platelets, and ECs. Simultaneous activation of PAR-1 and PAR-2 by thrombin is required to cause the chemokinetic effect. Thrombin-induced activation of PAR-2 could play a subtle role in signaling induced by the thrombin-PAR-1 complex and stimulate the motility of metastatic tumor cells [122].

Platelets can act as bridges between ECs and lymphocytes or MOs/MAs, and thrombin activation of platelets can enhance hematogenous metastasis [123]. In addition, platelet activation and fibrin formation are both important mechanisms by which tumor cell-related "procoagulant activity" promotes metastasis. Platelet activation induced by thrombin is another possible mechanism for metastasis, though some studies have shown that knockout of the PAR-1 and PAR-2 gene of mice does not affect thrombin signaling in mouse platelets but might attenuate signals in mouse ECs [124,125].

#### Factor XIII and fibrin

Factor XIII, fibrinogen, and fibrin contribute to the final step in the coagulation cascade. They are involved in some pathologic states, including solid tumor growth and the formation of ascites. Fibrin is formed by thrombin cleavage of fibrinopeptides A and B from fibrinogen, exposure of cryptic polymerization sites, and finally the cleavage of plasma fibrinogen to fibrin by thrombin. A more stable clot is formed when active factor XIII, a transglutaminase, is covalently cross-linked to fibrin α and γ chains. Factor XIII binds to integrin and might also exert antiapoptotic effects on ECs [16]. In our peritoneal transcript profile study, the factor XIII gene was upregulated in the peritoneum and stroma in the vicinity of the tumor [3]. The factor XIIIa subunit supports the adhesion of platelets mediated by α_IIb_β_3 _integrin [126] and fibroblasts mediated by α_v_β_3 _integrin and β_1_-containing integrins [127]. The α_v_β_3 _integrin, one of several integrins upregulated in the peritoneal transcript profile study [3], is present on vascular ECs and is a marker for angiogenesis. Factor XIII might also participate in angiogenesis. Factor XIIIa was shown to be a mediator of cell adhesion and an inhibitor of fibrin capillary tube formation in a dose-dependent manner [16].

Fibrin accumulation within tumors might result from increased permeability of tumor microvessels, which would lead to the leakage of plasma proteins, such as fibrinogen and plasminogen, and extravascular clotting and the cross-linking of fibrin to factor XIII [7]. In EOC ascites, high amounts of cross-linked fibrin degradation products have been identified [128]. VEGF could contribute to extravascular fibrin clotting in a tumor environment because it encourages leakage of plasma fibrinogen into extravascular spaces, where the fibrinogen clots [128]. Fibrin in the tumor or elsewhere in the peritoneum and serosa might help stimulate the new ingrowth of blood vessels. This fibrin gel matrix might also help provide the matrix that facilitates the ingrowth of MAs and fibroblasts and reorganizes the stroma, preparing it for tumor metastasis to the abdominal cavity [129,130]. Ascitic tumor cells as well as tumor cells derived from peritoneal and serosal surfaces can retain their viability in suspension cultures [131]. It is interesting that plasma exudates that contribute to ascites generally remain fluid and do not form an insoluble fibrin gel until removed from the patient [131]. Earlier studies showed that hyperpermeability of peritoneal lining vessels is required for ascites development in EOC tumors grown in murine models [132,133]. Cross-linked fibrin staining has not been observed in the peritoneal wall, diaphragm, mesentery, or bowel serosa of normal controls, but it has been detected in the peritoneum of ascitic tumor-bearing animals. Immunostaining revealed that adherent tumor cells were enmeshed in the fibrin meshwork binding them to each other and to peritoneal surfaces [129]. We also found that expression of phospholipase glutaminase A2, which mediates the first steps in the eicosanoid pathway, was increased in the peritoneum [3]. Leukotriene products on this pathway might contribute to capillary permeability and ascites formation.

### Fibrin and angiogenesis

Fibrin-induced neovasculation is based on clotting-related mechanisms that involve platelet activation and fibrin deposition. Cross-linked fibrin has been found in different human malignant tumors. It is present in the endothelium of angiogenic vessels of invasive cancer specimens but not in vessels of benign tumors [134]. Fibrin can bind to inflammatory cells or to tumor cells and is deposited around tumor cells as scaffolding that promotes further angiogenesis. Fibrinogen and fibrin fragments, such as fragment E, have been shown to stimulate angiogenesis both *in vitro *and *in vivo *[135]. The presence of the fibrin degradation product D-dimer is significantly associated with a poor prognosis in some cancer patients [136,137].

The binding of ECs to fibrin with the involvement of the adhesion molecule vascular endothelial cadherin may be necessary for capillary tube formation, a critical step in angiogenesis. The fibrin matrix that develops around tumors provides a provisional proangiogenic environment that supports vessel formation and stimulates EC proliferation and migration [138]. The fibrin matrix can promote a proangiogenic response by upregulating the expression of α_v_β_3 _integrin receptor to facilitate EC migration and capillary formation [139]. The α_v_β_3 _integrin provides survival signals to ECs during their interaction with fibrin. Tumor-containing tissue has revealed increased deposition of fibrin stimulated by VEGF-induced vessel leakiness to sustain the proangiogeneic environment [7]. The fibrin matrix also stimulates the production of VEGF, basic FGF, IGF1, and IL-8 to promote an autocrine procoagulant loop by inducing TF expression in ECs [61]. The expression of IGF1 and α_v_β_3 _integrin genes is upregulated in the peritoneum of EOC; therefore, fibrin may stimulate the production of these genes [3].

### Regulatory proteins in the coagulation cascade

Two important regulatory proteins in the coagulation cascade, serine proteinase inhibitor D1 (Serpins, also called heparin cofactor II – HCII) and endothelial protein C receptor (EPCR), were first detected as an upexpression in a study we conducted [3], which suggests that there is value in studying them further. Serpins are a protein superfamily of which many members possess potent activity as serine proteinase inhibitors [140]. Another very important family of serpins is antithrombins. Both HCII and antithrombins can inhibit the blood coagulation proteinase thrombin.

HCII is a 480-amino-acid, single-chain glycoprotein with a molecular weight of about 66 kDa that is synthesized by the liver and circulates in the plasma. The human HCII gene is located on chromosome 22q11, spans 15.8 kb, and includes 5 exons [141]. When HCII interacts with heparin and dermatan sulfate, the potential inhibition of thrombin can be increased by more than 1,000-fold *in vivo *[142]. HCII inactivates thrombin by forming a stable, bimolecular complex in plasma. HCII does not inhibit other proteases involved in coagulation or fibrinolysis. It can also participate in wound healing by regulating the mitogenic and chemotactic activities of thrombin [143]. Two recent clinical research studies demonstrated that HCII is a very effective factor in combating heart and vascular disease, which it does by inhibiting the action of thrombin [144,145]. HCII is inactivated after cleavage of its reactive site (Leu-444 through Ser-445) by elastase from leukocytes during the inflammation process without forming the stable thrombin-HCII complex [146].

HCII has a physiological role in the inflammation response. An active peptide from the amino-terminal region (corresponding to Asp-39 through Ile-66) with chemoattractant action for both neutrophils and MOs is proteolyzed by neutrophil elastase, and another leukocyte migration peptide is derived from dodecapeptide from Asp-49 through Tyr-60 of HCII. HCII-neutrophil proteinase products appear to have a role in the local inflammatory response [147]. Leukocytes and MOs/MAs are important cellular components of tissue injury, wound healing, and the microenvironment of EOC tumors [6]. The detection of upregulated HCII in the peritoneum and stroma of EOC patients is interesting because the degradation of HCII can produce a chemoattractant peptide for MAs [3]. MOs/MAs have multifunctional roles in EOC progression by releasing cytokines and chemokines, and HCII may help induce MA migration from the endothelium to extravascular tissue.

#### EPCR

EPCR is a 46-kDa type 1 transmembrane glycoprotein with two domains in the extracellular region that are homologous to the α1 and α2 domains of CD1/major histocompatibility complex class I molecules [148]. In humans, the EPCR gene is located on chromosome 20q11.2. Most EPCRs are expressed on the surface of ECs of vessels. A 43-kDa soluble EPCR has recently been found in the plasma of humans [149].

Downstream of coagulation, thrombin stimulates platelet aggregation, promotes coagulation by cleavage of fibrinogen, and fosters fibrin formation and activation of factor XIII. Thrombin is inhibited via the activated protein C (APC)-EPCR system. When thrombin binds to thrombomodulin on the surface of ECs, it activates protein C through EPCR to prevent coagulation. APC could inactivate factors V and VIII. The catalytic reaction for protein C activation by the thrombin-thrombomodulin complex is inefficient; only after combining with EPCR is protein C fully activated and does it acquire an anticoagulant role [150]. In addition to its anticoagulation role, APC inhibits thrombin-induced proinflammatory activity, such as platelet activation, cytokine-induced chemotaxis for MOs and neutrophils, and upregulation of leukocyte adhesion molecules. APC may prevent inflammation by downregulating TF and tumor necrosis factor-α (TNF-α), nuclear factor κB translocation, and cytokine signaling from MAs [151] and by inhibiting TNF-α-induced upregulation of cell surface leukocyte adhesion molecules [152]. APC may also protect the vasculature by blocking p53-mediated apoptosis in ischemic cerebral vasculature [153]. Because of its anti-inflammatory properties, APC is important in controlling serious infections. The apoptotic function of APC is independent of its anticoagulant function and requires EPCR as a cofactor but is mediated via PAR-1 [154]. When complexed with APC, membrane EPCR, which is expressed on the surface of ECs, could play a role in anticoagulation, inhibition of inflammation, and stimulation of cell proliferation (i.e., antiapoptosis) [155]. In contrast, soluble EPCR combines with protein C and APC with the same affinity but inhibits the anticoagulant activity of APC and interferes with the protective role of membrane EPCR and APC. Soluble EPCR may bind to and interfere with the function of neutrophil integrins, such as CD11b/CD18, which facilitates neutrophil adhesion to activated ECs and extravasation into extravascular tissue [156]. Soluble EPCR seems to amplify the inflammatory response [157,158]. High levels of soluble EPCR have been detected in sepsis [159] and in autoimmune disease [159]. Results from an animal model study showed that EPCR expressed on endothelium had a protective role in the cardiovascular system [160].

Only two studies relating to EPCR and cancer have been published [161,162]. Both studies were performed on cell lines. The membrane protein LMR42 was upregulated in most multidrug-resistant tumor cells. Expression cloning and sequence analysis showed LMR42 to be identical to EPCR. Elevated EPCR expression occurred in 47% of the primary tumor cell lines, including melanomas and renal and colon carcinomas. The authors of one of the studies concluded that elevated expression of EPCR may have a role in the resistant phenotype of multidrug-resistant tumor cells [161]. EPCR was also expressed on glioblastoma, leukemia, and most breast cancer cell lines; increased levels of APC activation were observed in tumor cells that express both EPCR and thrombomodulin [162]. Although the role of EPCR in cancer biology is poorly understood, it appears that EPCR may contribute to tumor progression. Our peritoneal transcript profile study showed high expression of EPCR on EOC peritoneum and its stroma [3]. PAR-1 is known to mediate a response in ECs, including the production of platelet-activating factor and chemokines (e.g., IL-8) and upregulation of adhesion molecules for neutrophils and platelets, which promote inflammation, thrombosis, and tumor progression [163]. However, the interaction of APC and PAR-1 is mediated by EPCR. At physiologic levels, APC is 10,000 times less potent than thrombin in this PAR-1-mediated pathway. When EPCR expression is upregulated, APC-PAR-1 signal transduction is significantly intensified, although it is still less potent than thrombin in this system [164].

The APC-EPCR interaction provides protection for ECs through its antiapoptotic effects. Because high EPCR expression occurs on tumors, EPCR might contribute to antiapoptosis in tumor cells. Finally, APC can stimulate PAR-1, whose activation depends on the EPCR concentration. A PAR-1-associated cellular response might contribute to tumor progression, possibly by producing IL-8, which stimulates tumor cell proliferation and metastasis.

### The connection between coagulation and inflammation in the EOC peritoneum

There is substantial evidence of infiltrating immune cells in EOC and the peritoneal environment. In a previous study, we showed that about 70% of T cells within EOC tumors were mononuclear cells [165]. Results from other researchers' experiments have suggested that the presence of these T cells is associated with an antigen-driven immune response [166-168]. However, there is little evidence to suggest there is a chronic cellular adaptive immune response *in vivo *because interferon γ message is absent or present at low levels [169]. Expression of the CD3ζ chain was absent or poor in another study [170]. A recent study showed longer overall and progression-free survival among EOC patients with intratumoral T cells than among those lacking these cells [168]. In a more recent study, CD4+CD25+ regulatory T cells present in EOC tumors appeared to be associated with poor patient survival. Regulatory T cells preferentially move to and accumulate in tumors and ascites but rarely enter the draining lymph nodes in later cancer stages [171]. Large numbers of MOs/MAs are also present in ascites, where they may make up 50% or more of mononuclear leukocytes, whereas the proportion that consists of T lymphocytes is usually less than 40% [165]. In recent preliminary studies, we found that pelvic peritoneal tissue biopsied from patients with advanced-stage EOC had a higher proportion of MOs/MAs than T cells even in the absence of tumor involvement (unpublished data).

Cytokines, chemokines, adhesion molecules, and components of the ECM may contribute to a tissue environment that supports tumor proliferation and invasion. Chemokines and certain cytokines may facilitate the migration of immune cells, including T cells and MAs, into the tumor environment (Tables 3 and 4). A network of CC (cysteines with no intervening amino acid) and CXC chemokines has been found in solid tumors and in ascites [172]. For example, CCL2, one of the ligands for CCR2, localizes to epithelial areas of the tumor [173], and its expression appears to correlate with the numbers of lymphocytes and MAs at this site [174]. CCL5 localizes with tumor-infiltrating leukocytes, and CCL5 concentration may be associated with CD8+ T cell infiltration. Chemokines CCL2, CC8, CCL4, CCL5, CCL8, CCL22, CXCL2, and CXCL12 have all been found in increased amounts in ascitic fluid from EOC patients, and the presence of CCL5 in ascitic fluid has been associated with the number of T cells in ascites [175].

**Table 3:** Cytokines involved in inflammation in ovarian cancer

**Cytokine**	**Effect**
TGF-β	Stimulates tumor cell attachment and invasion by upregulating plasminogen activator inhibitor type 1
	Deregulates expression of the major histocompatibility complex
	Deregulates costimulatory antigen expression by dendritic cells
	Suppresses Th1-Th2 cells and the conversion of pro-cytotoxic T lymphocytes to cytotoxic T lymphocytes
	Suppresses the proliferation response to antigen-presenting cells
	Inhibits natural killer and MA activation
IL-6	Upregulates tumor cell attachment
	Interferes with macrophage maturation in dendritic cells
	Inhibits cell proliferation through PI3K (phosphatidylinositol3-kinase)
	Suppresses Th1-Th2 transformation
IL-10	Deregulates expression of major compatibility complex on T cells
	Deregulates costimulatory antigen expression
	Suppresses cytotoxic T lymphocyte activation and Th1-Th2 transformation
	Inhibits interferon production
	Inhibits T cell production
VEGF	Increases neoangiogenesis, invasion, and metastasis
	Increases prevalence of ascites
FGF-2	Increases tumor cell invasion and metastasis
TNF-α	Increases adhesion molecule expression
	Induces tumor cell apoptosis
	Increases tissue factor expression
	Decreases the expression of thrombomodulin and EPCR

**Table 4:** Chemokines involved in ovarian pathogenesis

**Chemokine**	**Receptor**	**Cells targeted**	**Comments **
CCL22	CCR4	CD25^+^CD4^+ ^regulatory T cells	Forster tumor immune escape
CCL2	CCR2	Activated T cells, monocytes, and DC	Deregulate CD8^+ ^T cells and CD68^+ ^macrophage
CCL3	CCR2	Activated T, NK, MO, Eosinophils	Inflammatory cells migration
CCL4	CCR5	DC, MO, NK	Inflammatory cells migration
CCL5	CCR2	Activated T, NK, MO, Eosinophils	Inflammatory cells migration
CCL7	CCR2	Activated T, NK, MO, Eosinophils	Inflammatory cells migration
CCL18	Unknown	MOs/MAs	MA produced but not induced in EOC
CXCL8	CXCR1, CXCR2	Neutrophils and resting T cells	Angiogenesis, metastasis
CXCL12	CXCR4	Neutrophils, resting T cells, activated T and B cells, and macrophages	CXCR4 is preferentially expressed on EOC cells

Adhesion molecules may also be involved in the implantation of cancers cells onto the peritoneal lining. For example, E-cadherins and P-cadherins are expressed during EOC progression and may facilitate peritoneal invasion because mesothelial cells express cadherin-binding catenins [4]. Upregulation of discoidin domain receptor 1, claudin 3, and epithelial cell adhesion molecule occurs early in the development of EOC [176]. Soluble intracellular adhesion molecule 1 concentrations are elevated in patients with ovarian malignant tumors but do not correlate with clinical status[177].

Depending on their degree of expression in the tissue microenvironment, coagulation and inflammation might influence each other. Furthermore, components generated from both the coagulation cascade and an inflammatory response might be involved in heart disease, as recently reviewed [178-181]. Normal hemostasis is initiated when the blood vessels rupture, allowing blood cells to interact with extravascular cells and the ECM [182]. In one study, endotoxin, TNF-α, and IL-1α induced TF expression, primarily on MO/MA [58]. Blood clotting might thus be initiated when inflammatory cytokines and endotoxin induce the synthesis of TF on migrating leukocytes [183]. In this way, immune cells could help initiate the coagulation cascade through damaged tissue or cytokine production [184] (Table 5).

**Table 5:** Procoagulant effects of inflammation products

**Product **	**Procoagulant effect**
Leukocytes or MAs	Activate platelets Induce microvascular occlusion Release neutrophil elastase Induce thrombomodulin release from the endothelium Inactivate antithrombin
Complements	Increase procoagulant factors production Provide membrane surfaces Augment TF expression
Inflammatory Cytokines	Increase TF production Decrease thrombin, EPCR, and protein S levels Increase C-reactive protein production Increase complement activation Increase platelet count and reactivity
Chemokines	Activate platelet aggregation and adhesion Attract more leukocytes

The complement system may also contribute to hemostasis by activating the membrane attack complement pathway, thereby contributing to phosphatidylserine expression on the outer membrane of cells [185]. Expression of phosphatidylserine on the outer cell membrane is necessary for the effective initiation and amplification of the coagulation cascade. Our microarray analysis of the peritoneum of EOC patients showed increased expression of the C2 component [3]. In contrast, expression of complement HF1, which blocks the membrane attack complement pathway, was increased and might have shifted the complement activation pathway toward a proinflammatory response with the recruitment of MO/MA. MO/MA are also an important source of complement components.

Chemokines can influence coagulation by activating platelets indirectly. Three main chemokines are involved in this process, CXCL12 (the ligand for CXCR4), CCL17 (the ligand for CCR4), and CCR22 (the ligand for CCR4) [186]. Platelets are an important link in the inflammation and coagulation processes. Inflammatory mediators, cytokines, and chemokines do not increase platelet production, but the platelets generated are more thrombogenic and thus are more sensitive to platelet agonists such as thrombin. Platelets contain high concentrations of the proinflammatory mediator CD40 [187] and certain chemokines [188]. When CD40 and these chemokines are released, they can induce TF synthesis and increase the production of inflammatory cytokines such as IL-6 and IL-8. Of the naturally occurring anticoagulants, protein C is the most negatively influenced by inflammation. Thrombomodulin and EPCR are both downregulated by inflammatory cytokines such as TNF-α [189] and IL-1 [190].

Thrombin has a variety of effects on cells that can enhance the inflammatory response. For example, it augments leukocyte adhesion and activation, which amplifies the inflammatory response, and induces endothelium and platelet activation. The activation of platelets can produce cytokines (IL-6) and chemokines (IL-8), which are involved in inflammatory responses [186]. Thrombin also acts as a direct mitogen for fibroblasts, inducing PDGF and TGF from platelets and ECs. PDGF and TGF-β are important cytokines for stimulating neovessel formation, and TGF-β may contribute to an environment conducive to tumor escape from immune system surveillance. Notably, PDGF and TGF-β 3 receptor expression were increased in our peritoneal transcript profile study [3]; this finding provides additional evidence that thrombin could foster tumor cell growth and proliferation via PDGF and TGF release from platelets and ECs.

### Anticancer therapy based on targeting the coagulation cascade

Coagulation factors have a profound effect on tumor cell behavior in both *in vivo *and *in vitro *studies. These factors could enhance tumor cell proliferation, invasion, angiogenesis, and metastasis. Hence, targeting activated coagulation factors might provide a viable cancer treatment strategy.

Heparins are the most extensively used anticoagulants in clinics. In blood coagulation, unfractionated heparin and low-molecular-weight heparins potentiate the activity of antithrombin III, thus inhibiting the activation of coagulation factors II and X [191]. They also release TFPI, a physiologic inhibitor of the TF pathway that prevents pulmonary embolism and is used to treat deep vein thrombosis [192]. Retrospective and meta-analytic studies of deep vein thrombosis treatment have shown longer survival among cancer patients with thrombosis who were treated with unfractionated heparin and low-molecular-weight heparins than among patients treated without heparin [193-198]. Thus, the use of anticoagulants might allow them to live longer.

There is a higher incidence of venous thromboembolism in patients with melanoma or small cell lung cancer. In 2003, a pilot study of enoxaprin, a low-molecular-weight heparin for the treatment of advanced melanoma was reported [199], but the clinical outcome was not clear. Then, a phase II trial of combination chemotherapy and anticoagulant therapy (docetaxel plus enoxaparin) was performed in chemotherapy-naive patients with metastatic non-small cell lung cancer [200]. The median time to progression was 5 months, and the median survival time was 11 months. The most frequent toxic effects were neutropenia and asthenia; no clinically significant bleeding or thrombotic events were observed. Treatment was well tolerated in patients with advanced small cell lung cancer, and the results suggested that enoxaparin could prolong the time to disease progression [200]. Both the oral anticoagulant warfarin and unfractionated heparin have been shown to prolong survival time among patients with small cell carcinoma [201].

In 2004, a prospective, randomized, controlled trial enrolled 385 cancer patients into two arms, placebo and dalteparin (another low-molecular-weight heparin). Types of cancer included breast, colorectal, ovarian, and pancreatic [202]. Thirty-four percent of the dalteparin group and 31% of the placebo group received chemotherapy alone; 8% of each group received radiation therapy alone. Estimated overall survival at 1, 2, and 3 years did not differ significantly between groups overall. However, the estimated overall survival among patients with a better prognosis at enrollment (55 patients in the dalteparin group and 47 patients in the placebo group) was significantly longer in the dalteparin group at 2 years (78% and 60%; P = 0.03) and at 3 years (55% and 36%; P = 0.03). In 2005, another study confirmed these antineoplastic effects of dalteparin [203]. During the 12-month follow-up period of the study, 602 patients with solid tumors and venous thromboembolism were randomly assigned to a dalteparin or coumarin-derivative treatment group. Among patients without metastatic disease, the probability of death at 12 months was 20% in the dalteparin group compared with 36% in the oral anticoagulant group (P = .03). In patients with metastatic cancer, no difference was observed in mortality between the treatment groups (72% and 69%, P = .46). However, the observed effects of dalteparin on survival were statistically significantly different between patients with and without metastatic disease (P = .02). Clinical trials are warranted to investigate these findings. The exact mechanism by which heparin mediates antitumor or antimetastatic activity is unknown but also merits further study.

## Conclusion

Factors from the extrinsic and intrinsic coagulation cascades play complex and important roles in cancer progression by promoting blood clotting; in the microenvironment of cancer (e.g., ECs, platelets, fibroblasts, leukocytes, and the ECM), altered gene expression affects key intracellular signaling events. Certain signaling pathways may facilitate thrombus formation in the peritumoral environment and promote localized angiogenesis. In our study of transcript profiles of the peritoneum, several factors that are part of the intrinsic coagulation cascade were overexpressed at the transcript level in the peritoneum in the vicinity of but beyond the periphery of the tumor [3]. Overall, the extrinsic and intrinsic pathways favor clot formation. Thus, products of the peritoneal environment, which include chemokines, cytokines, and coagulation factors or their receptors, are evolving as potential targets for biologic therapy or *in vivo *diagnostic tools. The number of humanized monoclonal antibodies applicable to cancer treatment is increasing as more targets are discovered. Other approaches to treatment might include inhibitory oligonucleotides packaged to avoid degradation and small molecules that block specific pathways. Retrospective studies have shown that antithrombotic therapy may be associated with a lower incidence of certain types of cancer [204]. Clinical trials need to be expanded to target the production of certain coagulation factors or receptors in the coagulation cascade of advanced ovarian cancer. Such studies may reveal new approaches for chemoprevention or combination therapy that include chemotherapy to control tumor proliferation, angiogenesis, and metastasis.
